# Reviewed article: Nerbass FB, Hanauer MA, Silva IF, Costa TF, Silva RM, Fabre L, Vieira MA, Calice-Silva V. Profile, follow-up adherence and progression of people with advanced chronic kidney disease in specialized care centers: a retrospective study, Santa Catarina state, Brazil, 2021-2024. Epidemiol Serv Saude. 2026:35;e20250690.

**DOI:** 10.1590/S2237-96222026v35e20250690.a

**Published:** 2026-04-10

**Authors:** Janielle Ferreira de Brito Lima

**Affiliations:** 1 Universidade Federal do Maranhão, São Luís, MA, Brazil

## Peer review A

## Questionnaire

Does the manuscript contain new and significant information to justify publication? Yes

Does the Abstract (Summary) clearly and accurately describe the content of the article? Yes

Is the problem significant and concisely stated? Yes

Are the methods described comprehensively? Yes

Are the interpretations and conclusions justified by the results? Yes

Is adequate reference made to other work in the field? Yes

## Manuscript Structure

Length of article is: Adequate

Number of tables is: Adequate

Number of figures is: Adequate

Please state any conflict(s) of interest that you have in relation to the review of this paper. None

Interest: Excellent

Quality: Good

Originality: Good

Overall: Good

Recommendation: Minor Revision

## Comments

O manuscrito apresenta um estudo multicêntrico retrospectivo de relevância significativa para a saúde pública. Aborda o perfil, a adesão ao seguimento e a evolução de pacientes com doença renal crônica avançada em centros especializados do estado de Santa Catarina. O tema é atual, pertinente e alinhado às prioridades do SUS, especialmente diante do envelhecimento populacional e da crescente prevalência de doenças crônicas no país. A redação está clara, os objetivos estão bem delimitados, e os métodos utilizados são coerentes com a proposta do estudo. Os resultados são apresentados de forma organizada, e a discussão está fundamentada, com articulação adequada com a literatura científica nacional e internacional. O estudo contribui para o conhecimento sobre a efetividade do acompanhamento especializado de pacientes com DRC em estágios avançados, demonstrando boa taxa de adesão ao seguimento e estabilidade da função renal ao longo de um ano. Recomenda-se a aceitação do artigo, mediante pequenos ajustes de forma e conteúdo, que estão descritos a seguir.

- No resumo, corrigir o termo “doença renal crônia” para “doença renal crônica” na seção de descritores.

- Revisar o dado apresentado sobre a TFG final [“22 (18-7)”], pois há provável erro tipográfico no intervalo interquartil (IIQ).

- Corrigir pequenos erros de digitação identificados na introdução, como “especiailzado”.

- Incluir na introdução breve descrição dos critérios clínicos para a classificação dos estágios 4 e 5, já que isso fundamenta o recorte da amostra.

- Explicitar os critérios de exclusão utilizados nos métodos.

- Justificar a escolha do intervalo entre 10 e 14 meses para caracterizar o seguimento de 12 meses.

- Esclarecer se houve padronização entre os centros quanto aos protocolos de atendimento e coleta de dados.

- Verificar a consistência do IIQ da TFG na Tabela 2, que parece ter erro de digitação.

- Na Figura 1, rever o título.

- Padronizar o uso da sigla DRC ao longo de todo o texto.

- Na discussão sugiro reforçar as limitações do delineamento retrospectivo e ausência de grupo controle.

- Não parece adequado atribuir causalidade direta entre o acompanhamento e a preservação da função renal. Rever.

- Seria oportuno enfatizar a importância da análise dos fatores organizacionais que favoreceram a boa adesão ao seguimento (ex. transporte, agendamento imediato, curto tempo de espera) na discussão.

- As poderiam incluir recomendações para o SUS, reforçando o papel da atenção especializada na prevenção da progressão da DRC.

